# *Bifidobacterium animalis* subsp. *lactis* BPL1™ and Its Lipoteichoic Acid Modulate Longevity and Improve Age/Stress-Related Behaviors in *Caenorhabditis elegans*

**DOI:** 10.3390/antiox12122107

**Published:** 2023-12-13

**Authors:** Ferran Balaguer, Marta Barrena, María Enrique, Miren Maicas, Beatriz Álvarez, Marta Tortajada, Empar Chenoll, Daniel Ramón, Patricia Martorell

**Affiliations:** Archer Daniels Midland, Nutrition, Health & Wellness, Biopolis S.L. Parc Científic Universitat de València, C/Catedrático Agustín Escardino Benlloch, 9, 46980 Paterna, Spainmarta.barrenacastillo@adm.com (M.B.); maria.enrique@adm.com (M.E.); marta.tortajadaserra@adm.com (M.T.); maria.chenoll@adm.com (E.C.);

**Keywords:** *Bifidobacterium animalis* subsp. *lactis*, BPL1, LTA, aging, *C. elegans*, probiotic, postbiotic

## Abstract

Life expectancy has increased globally in recent decades, driving interest in maintaining a healthy life that includes preservation of physical and mental abilities, particularly in elderly people. The gut microbiome becomes increasingly perturbed with aging so the use of probiotics can be a strategy for maintaining a balanced gut microbiome. A previous report showed that *Bifidobacterium animalis* subsp. *lactis* BPL1™ induces through its lipoteichoic acid (LTA) fat reduction activities via the insulin/IGF-1 signaling pathway. Here, we have delved into the mechanism of action, eliminating alternative pathways as putative mechanisms. Furthermore, we have identified that BPL1™, its heat treated form (BPL1™ HT) and its LTA prolong longevity in *Caenorhabditis elegans (C. elegans)* in an insulin/IGF-1-dependent mechanism, and its consumption improves the oxidative stress response, gut permeability and protection against pathogenic infections. Furthermore, positive effects on *C. elegans* stress-related behaviors and in the Alzheimer’s Disease model were found, highlighting the potential of the strain in improving the cognitive functions and proteotoxicity in the nematode. These results indicate the pivotal role of the IGF-1 pathway in the activity of the strain and pave the way for potential applications of BPL1™, BPL1™ HT and its LTA in the field of longevity and age-related markers.

## 1. Introduction

Life expectancy has increased globally by more than 6 years in the period from 2000 to 2019 while healthy life expectancy without disabilities has not improved at the same level [1]. In recent years, the role of the human gut microbiome has been highlighted as key to support a healthy status while it is well known that this gut microbiome becomes increasingly perturbed with aging, so the use of probiotics can be a strategy for maintaining a balanced gut microbiome later on in life [2]. In this context, discovering and understanding the mechanisms of action of probiotics and postbiotics that support healthy aging is key to developing novel solutions that support healthy aging. Among probiotics, *Bifidobacterium* sp. strains have been shown to be effective in longevity, antioxidative stress, ameliorating the gut barrier dysfunction or improving metabolic health and its associated markers in pre-clinical models [3,4,5,6,7,8]. We have previously shown the effectiveness of the *Bifidobacterium animalis* subsp. *lactis* (BPL1™) strain, its heat-treated form (BPL1™ HT) and its lipoteichoic acid (LTA) on fat reduction when using the animal model *Caenorhabditis elegans* (*C. elegans*) [9,10]. This nematode has been widely used to identify regulators of fat metabolism due to the high genetic conservation regarding pathways regulating fat metabolism and storage [11]. LTA is one of the first key probiotic effector molecules reported in pre-clinical modulation of obesity [10]. Additionally, the functionality of the BPL1™ and BPL1™ HT was also positively assessed in pre-clinical studies with Zücker rats and Wistar rats under a cafeteria diet as well as in clinical studies with human obese volunteers [12,13,14]. Interestingly, BPL1™ was also shown to be effective in the reduction in adiposity in children with Prader–Willi syndrome, with additional modest improvements in some parameters related to mental well-being [15].

In our previous work, we demonstrated that the pre-clinical anti-obesity effect of the probiotic BPL1™, the postbiotic BPL1™ HT and the purified LTA from BPL1™ was dependent on the insulin/IGF-1 signaling pathway [9,10]. In *C. elegans*, the key regulatorsof this pathway are the insulin-like growth factor 1 (IGF-1) receptor DAF-2 and the FOXO transcription factor DAF-16 [16]. Additionally, different studies have demonstrated that the insulin/IGF-1 signaling pathway acts in combination with other pathways, such as the p38 MAPK and the JNK-1/DAF 16 pathways [16]. The insulin/IGF-1 signaling pathway is an evolutionarily conserved pathway, which in *C. elegans*, also plays a pivotal role in pathogen defense, growth, development, longevity and behavior [17]. Thus, BPL1™, BPL1™ HT and its purified LTA might play a role in modulating a wide range of functions.

In this study, we first aimed to further delve into the mechanisms of action of BPL1™, BPL1™ HT and LTA in a pre-clinical model of obesity. Also, we characterized the role of BPL1™, BPL1™ HT and LTA in promoting longevity in *C. elegans*. We found that insulin/IGF signaling is the central pathway targeted by BPL1™, BPL1™ HT and LTA to reduce lipid accumulation, eliminating other putative pathways. Also, we found that BPL1™, BPL1™ HT and LTA showed positive effects on longevity and age-related markers such as oxidative stress, intestinal integrity, behavior and cognitive scores in an Alzheimer’s Disease model in *C. elegans*. These findings in the *C. elegans* model suggest that consumption of BPL1™, BPL1™ HT and LTA could have promising prospects to support healthy aging, and further research in human subjects is warranted to support the evidence.

## 2. Material and Methods

### 2.1. C. elegans Strains and Maintenance Conditions

*Caenorhabditis elegans* N2, a Bristol (wild-type) strain and the mutant strains GR1307 *daf-16(mgDf50)* I; CB1370 *daf-2(e1370)* III; KU25 *pmk-1(km25*) IV; VC8 *jnk-1(gk7)* IV; TJ356 *zIs356[daf-16p::daf-16a/b::GFP+rol-6(su1006)]* IV and DA650 *npr-1(g320)* were provided by the *Caenorhabditis* Genetic Center (CGC), University of Minnesota (Minneapolis, MN, USA).

The nematodes were maintained on Nematode Growth medium (NGM) plates with *Escherichia coli* OP50 as a normal diet. Worms were synchronized by isolating eggs from gravid adult nematodes grown at 20 °C on fresh NGM plates with OP50.

For paralysis assays, the transgenic strain CL4176 (*smg-1ts*; [pAF29 (*myo-3/Ab1–42/let* UTR) + pRF4 (*rol-6(su10069)*)]) was provided by Dr. Christopher D. Link. This strain was routinely propagated on OP50 plates at 16 °C.

### 2.2. Bacterial Strains and Culture Conditions

*Bifidobacterium animalis* subsp. *lactis* BPL1™ (CECT 8145) was routinely grown in an Man Rogosa Sharpe (MRS) medium (Peptone from casein tryptic digest at 10 g/L; D-glucose at 20 g/L; MnSO_4_ at 0.05 g/L (obtained all from Merck, Darmstadt, Germany); meat extract at 10 g/L; yeast extract at 10 g/L (both from Condalab, Madrid, Spain); K_2_HPO_4_ at 2 g/L; di-ammonium hydrogen citrate at 2 g/L; sodium acetate at 5 g/L; MgSO_4_ at 0.2 g/L; Tween 80 at 1 g/L (from Scharlab, Sentmenat, Spain); supplemented with cysteine (Sigma, St. Louis, MO, USA, 0.05% wt/vol), MRS-Cys, for 18 h at 37 °C in an anaerobiosis atmosphere generated by the GasPakTM EZ Anaerobe Container System (BD, Franklin Lakes, NJ, USA), as described elsewhere [9,10].

For BPL1™ heat inactivation, the same protocol applied in previous studies was followed [10].

The *Escherichia coli* OP50 strain was routinely cultured in a Luria-Bertani (LB) broth medium (Bacto-tryptone at 10 g/L (Condalab, Madrid, Spain); Bacto-yeast at 5 g/L (Condalab, Madrid, Spain); NaCl at 5 g/L (VWR, Leuven, Belgium) at 37 °C for 18 h and then was used for the seeding of NGM plates.

*The salmonella enterica* subsp. *enterica* ATCC 14028 strain (serovar Typhimurium) was grown on aLB medium and the *Staphylococcus aureus* ATCC 25923 strain was cultured on Tryptic Soy Broth (TSB) aerobically.

### 2.3. Preparation of LTA from BPL1™

Lipoteichoic acid from BPL1™ was isolated and purified from BPL1™ cultures as previously described [10]. Briefly, cells were harvested and mechanically disrupted. The pellet cell membrane material was mixed with an equal volume of n-butanol (Acros Organics, Geel, Belgium) and stirred for 45 min at 37 °C. After centrifugation (12,000× *g*, 90 min at 4 °C), a lower aqueous phase was collected. The sample was freeze-dried and subjected to hydrophobic interaction chromatography (HIC) on an octyl-Sepharose (Cytiva Sweden AB, Uppsala, Sweden) column using a linear gradient from 15% to 65% *n*-propanol (Sigma, St. Louis, MO, USA) in a 0.05 M citrate buffer at pH 4.7. Phosphate-containing fractions (molybdenum blue test detected) were pooled and lyophilized. For the experiments with *C. elegans*, we prepared the LTA stock solution at a final concentration of 1mg/mL with sterile MilliQ water and added to the plates at each specific concentration.

### 2.4. Fat Reduction Assays

The *C. elegans* fat content under the different feeding conditions was measured using Nile Red (Sigma, St. Louis, MO, USA) staining and subsequent fluorescence quantification following the protocol previously described [9]. Nematodes were fed with BPL1™, BPL1™ HT and LTA purified from BPL1™. In the case of live and heat-treated BPL1™, the cells were added directly to the surface of NGM plates with OP50 at a dose of 10^8^ cells/plate. The purified LTA isolated from BPL1™ was added to the surface of NGM plates at a final concentration of 10 µg/mL. As a positive control of the assay, the anti-obesity drug Orlistat (Sigma, St. Louis, MO, USA) was used at a final concentration of 6 µg/mL [18]. Two independent experiments were carried out, analyzing 120 worms/condition.

### 2.5. Lifespan Assays

For lifespan assays, synchronized nematodes of N2 or the corresponding mutant strains were cultured until the L4 stage at 20 °C. Then, the worms were transferred to NGM plates already seeded with the *E. coli* OP50 strain, or to NGM plates with a lawn of BPL1™. In the case of BPL1™ HT, cells were added to a final concentration of 10^9^ and 10^10^ cells/plate. The LTA from BPL1™ was assessed at different doses (0.1, 0.5, 1, 5, 10, 25 and 50 µg/mL) with the wild-type strain N2. The plates were maintained at 20 °C. The number of live nematodes was scored until 100% of the population was dead. Two independent experiments were conducted for each condition with 100/200 worms analyzed.

### 2.6. Oxidative Stress Assay

The antioxidant activity of LTA from BPL1™ and BPL1™ HT was assessed in *C. elegans* by means of the previously described assay using the wild-type N2 strain [19]. Briefly, egg-synchronized worms were placed in NGM plates with four different doses of LTA from BPL1™ (1, 5, 10 and 25 µg/mL). An oxidative stress was applied with the addition of H_2_O_2_ (Merck, Darmstadt, Germany) (2 mM) during 5 h and the viability of the worms was measured after this time. Vitamin C (Sigma, St. Louis, MO, USA) (10 µg/mL) was used as a positive control of the assay. The experiment was carried out in duplicate, analyzing 100 worms per condition.

### 2.7. Infection Assays

Infection assays were performed as previously described with minor modifications [20,21,22]. Wild-type synchronized eggs were seeded in NGM plates with the *E. coli* OP50 strain or with different doses of purified LTA from BPL1™ (1, 5 and 10 µg/mL), BPL1™ or BPL1™ HT at 10^8^ cells/plate. Once the worms reached the young adult stage, they were transferred to the infection plates containing a lawn of the respective pathogen (*S. aureus* ATCC 25923 strain or *S. enterica* subsp. *enterica* serovar Typhimurium ATCC 14028 strain). A negative control condition without infection (NGM medium with *E. coli* OP50 strain) and a positive control condition of infection with only the corresponding pathogen were included. Worms were maintained at 25 °C in the different conditions and scored for survival during 5–10 days. Worms were counted as alive or dead by stimulation with the platinum stick. Two independent assays were carried out with a total of 100 worms analyzed per condition.

### 2.8. Gut Barrier Integrity

Age-synchronized nematodes of the wild-type strain N2 were obtained and maintained in NGM plates or NGM plates supplemented with the strain BPL1™ (10^7^ and 10^8^ cfu/plate), BPL1™ HT (10^7^ and 10^8^ cells/plate) or LTA from BPL1™ (10 µg/mL and 1 µg/mL) at 20 °C. To induce intestinal permeability, L4 larvae were exposed to H_2_O_2_ (0.8 mM) for 24 h [23]. A control condition without larval damage was also included. To evaluate the intestinal permeability in H_2_O_2_-exposed nematodes, Nile Red staining (0.05 g/mL) was used [24]. A total of 30 worms were randomly selected from each condition and observed in a fluorescence stereomicroscope, Nikon SMZ18 (Tokyo, Japan), equipped with NIS-ELEMENT AR image software(5.02.00). Results are shown as the percentage of fluorescence in each treatment with respect to the H_2_O_2_-treated nematode population. Two independent assays were performed.

### 2.9. DAF-16 Nuclear Translocation

The intracellular localization of DAF-16 was assessed with the *C. elegans* TJ356 reporter strain, following the protocol previously described [25]. Briefly, worms were synchronized in NGM plates with BPL1™ (10^8^ cfu/plate), BPL1™ HT (10^8^ cells/plate) or LTA from BPL1™ (10 µg/mL) until they reached the young adult stage. Then, worms were transferred to plates with 1% agarose (Condalab, Madrid, Spain) and 0.1% sodium azide (Sigma, St. Louis, MO, USA) and nuclear translocation was measured using the Nikon SMZ18 (Tokyo, Japan) fluorescence stereomicroscope equipped with NIS-ELEMENT AR image software (5.02.00). Fifty worms per condition were assessed in two different experiments.

### 2.10. Behavioral Assays

#### 2.10.1. Avoidance Behavior

Octanol avoidance was used as an anxiety-related behavior assay and the experiments were carried out using the “smell-on-a-stick” assay described elsewhere [26]. For the assays, the blunt end of a hair of a paintbrush (Loew-Cornell (Teaneck, NJ, USA) 9000 Kolinsky 7 paintbrush) taped to a Pasteur pipette was immersed in freshly prepared 30% octanol (Merck, Darmstadt, Germany) in EtOH (Scharlab, Sentmenat, Spain) (vol/vol) and placed in front of a forward-moving nematode. Control condition nematodes were maintained in NGM with OP50 *E. coli*. And were incubated for 20 min in fresh plates before testing. For the anxiety condition, nematodes were incubated for 20 min in NGM plates without the *E. coli* OP50 strain. This food deprivation induces anxiety-related behavior due to decreased levels of serotonin [26]. To evaluate treatments, wild-type nematodes were age-synchronized in NGM plates supplemented with BPL1™ (10^8^ cfu/plate), BPL1™ HT (10^8^ cells/plate) or its LTA (10 µg/mL). At the young adult stage, nematodes were transferred to NGM plates with each treatment but no food for 30 min. Afterwards, worms were placed in NGM plates without OP50 and with each treatment and tested 20 min later. The amount of time that worms took to move backward was measured. Two independent assays were carried out with 80 worms analyzed per condition.

#### 2.10.2. Reduction in Stress

In this experiment, the nematode’s exploratory behavior was analyzed after the addition of the psychostimulant drug Denubil (Pierre Fabre, Barcelona, Spain). The nematodes were incubated in NGM plates with BPL1™ (10^8^ cfu/plate), BPL1™ HT (10^8^ cells/plate) or its LTA (10 µg/mL) and transferred to NGM plates supplemented with the psychostimulatory drug until they reached the young adult stage. These nematodes were picked and moved to the assay plates.

The assay plates (NGM seeded with *E. coli* OP50 strain) were divided into three concentric areas measuring <0.9 cm, 0.9–1.8 cm and 1.8–2.8 cm to the center point. The nematodes were placed in the middle of the plate and then scored for their position after 2 min. These assays were carried out in duplicate with 60 worms per condition.

### 2.11. Paralysis Assays (Alzheimer’s Disease C. elegans Model)

Paralysis experiments were performed using the transgenic *C. elegans* strain CL4176 as previously described [27]. Briefly, nematodes were synchronized by isolating eggs from gravid worms at 16 °C in NGM plates (control condition) and NGM plates with an overnight lawn of BPL1™, BPL1™ HT (1 × 10^8^ cells/plate) or different doses of LTA from BPL1™ (0.05, 0.1, 1 and 10 µg/mL). As a positive control, the *G. biloba* extract EGb 761^®^(100 μg/mL) (Tanakene, Ipsen Pharma, S.A., Sant Feliu de Llobregat, Spain) was also added on NGM plates. The induction of the muscle-specific Aβ1–42 transgene expression was carried out by raising the temperature from 16 °C to 25 °C, starting 48 h after the synchronization and maintained for 24 h, when the paralysis was scored. Paralysis in induced worms was compared with non-induced worms (maintained at 16 °C until the end of the paralysis assay). Experiments were carried out in duplicate, analyzing 100 worms per condition.

### 2.12. Statistical Analysis

Results are shown as the mean ± standard deviation. For fat reduction, oxidative stress and gut permeability assays, data were analyzed by one-way ANOVA, using Tukey’s multiple comparison test. A Log Rank *t*-test was applied for the lifespan, infection and body paralysis assays. The octanol avoidance assay was analyzed using Student’s *t*-test and cholinergic stress assays were analyzed using two-way ANOVA. All the analyses were performed with GraphPad Prism 9 software (GraphPad Software Inc., San Diego, CA, USA), adjusting the level of significance at 5%.

## 3. Results

### 3.1. Fat Reduction Effect of BPL1™, Heat-Treated BPL1™ and LTA from BPL1™ Requires the Insulin-like Signaling Pathway (IGF-1) and Not the MAPK Pathway in C. elegans

Previous pre-clinical studies with BPL1™ live cells have reported that its fat reducing effect is mediated by the IGF-1 pathway [9]. Furthermore, BPL1™ HT and purified LTA from BPL1™ also require this signaling pathway to exert the activity [10].

**Figure 1:**
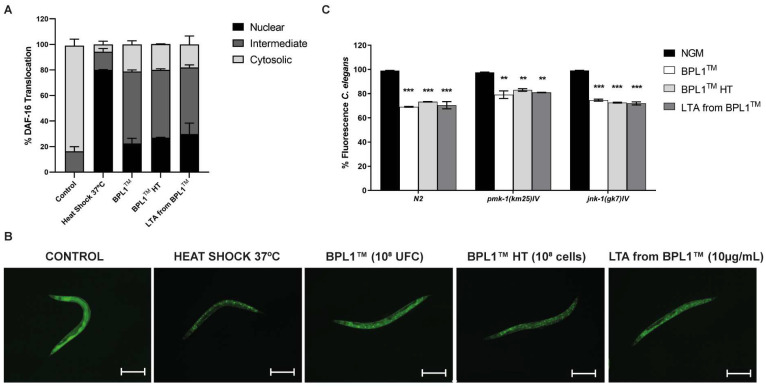
BPL1™, BPL1™ HT and LTA from BPL1™ exert fat reduction activity through IGF-1. (**A**) Percentage of nematodes with the different cellular localization of DAF-16 after feeding with BPL1™ (10^8^ cfu/plate), BPL1™ HT (10^8^ cells/plate) and LTA from BPL1™ (10 µg/mL). (**B**) Representative images of the DAF-16 nucleus translocation of nematodes fed with BPL1™, BPL1™ HT and LTA from BPL1™. Image taken with a Nikon-SMZ18 Fluorescence Stereomicroscope (Scale bar: 250 µm). (**C**) Percentage of Nile Red fluorescence of N2 wild-type worms of *pmk-1(km25)* and *jnk-1(gk7)* mutant strains fed with BPL1™ (10^8^ cfu/plate), BPL1™ HT (10^8^ cells/plate) or LTA from BPL1™ (10 µg/mL). Nile Red staining fluorescence was quantified at young adult stage. Orlistat (6 µg/mL) was used as a positive control. Data are mean ± SD and were calculated from two independent experiments (*n* = 120/condition for fat deposition, *n* = 50/condition for DAF-16 nuclear translocation). ** *p* < 0.01, *** *p*< 0.001. One-way ANOVA was applied.

To further determine the mechanism of action, first we checked the activation of DAF-16 by a nuclear translocation assay using the TJ356 reporter strain, in the presence of BPL1™, BPL1™ HT or LTA from BPL1™. By fluorescence microscopy, we observed an increase in nuclear DAF-16 in nematodes fed with the three ingredients when compared with control nematodes with the *E. coli* OP50 strain standard diet (Figure 1A,B). This increase revealed that BPL1™, BPL1™ HT as well as LTA from BPL1^TM^ promote the activation of DAF-16, which is required for their functionality.

Furthermore, we explored other pathways that may be involved in the functionality of BPL1™. For this purpose, fat reduction activity of BPL1™, BPL1™ HT and LTA from BPL1™ was evaluated in *C. elegans* mutant strains through alternative pathways. Besides acting in the IGF-1 pathway, DAF-16 is located downstream on the mitogen-activated protein kinase pathway (MAPK) [16]. Two protein kinases were selected, PMK-1/p38 and JNK-1, as representatives of the MAPK family, which can be activated by environmental stress factors and ultimately activate the nuclear localization of DAF-16 [28,29].

The fat reduction assays showed that BPL1™, BPL1™ HT and LTA from BPL1™ maintain their activity in *pmk-1(km25)* and *jnk-1(gk7) C. elegans* mutant strains (Figure 1C). These results indicate that BPL1™ does not require either PMK-1/p38 or JNK-1 pathways to exert its fat reduction activity, acting through DAF-16 via the IGF-1 pathway. Moreover, the results showed a good correlation between the BPL1™ live cells, its heat-treated form and its LTA.

### 3.2. BPL1™, Heat-Treated BPL1™ and LTA from BPL1™ Prolong Lifespan via IGF-1 and Protect against Age-Related Phenotypes in C. elegans

Having demonstrated that the IGF-1 pathway is the main target of BPL1™, BPL1™ HT and LTA from BPL1™, and the fact that IGF-1 has emerged as a major regulator of the aging process [30], we hypothesized that the probiotic and postbiotic could be effective on longevity.

**Figure 2:**
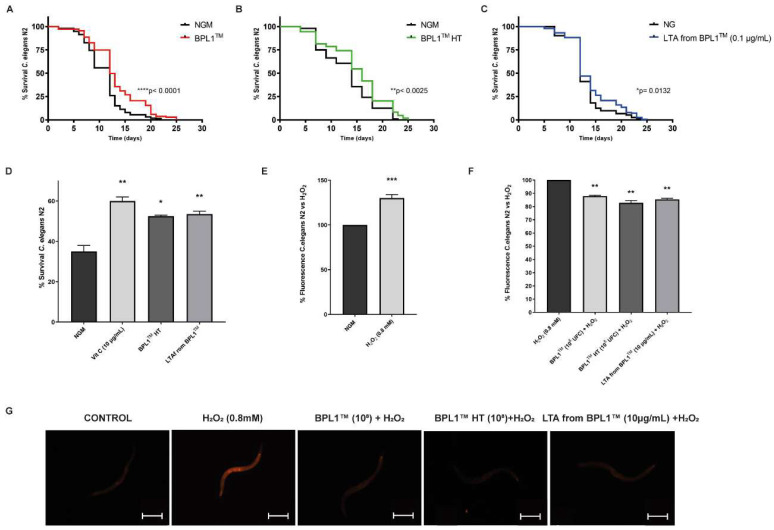
Positive effect of BPL1™, BPL1™ HT and LTA from BPL1™ on *C. elegans* lifespan and other age-related phenotypes. Survival rate of N2 wild-type worms fed with BPL1™ alive cells (**A**), BPL1™ HT (**B**) and LTA from BPL1™ (**C**). Curve comparisons vs. *E. coli* OP50 are indicated (*p*-values). Two independent experiments were performed (*n* = 100–200/condition). Log Rank *t*-test was applied. (**D**) Survival rate of *C. elegans* N2 wild-type strain fed with BPL1™ HT (10^8^ cells/plate) and LTA from BPL1™ (5 µg/mL) after acute oxidative stress. NGM was used as a control feeding condition. * *p* < 0.05, ** *p* < 0.01. Data correspond to two independent experiments (*n* = 100/condition). One-way ANOVA was applied. (**E**) Percentage of fluorescence of nematodes without intestinal damage (control) or worms with damage provoked with 0.8 mM of H_2_O_2_. *** *p* < 0.005. Two independent experiments were performed (*n* = 60/condition). (**F**) Percentage of fluorescence of nematodes damaged with 0.8 mM of H_2_O_2,_ fed with BPL1™ (10^8^ cfu/plate), BPL1™ HT (10^8^ cells/plate) or LTA from BPL1™ (10 µg/mL). ** *p* < 0.01. Values are the average of two independent assays (*n* = 60/condition). One-way ANOVA was applied. (**G**) Representative images of fluorescence nematodes treated with 0.8 mM of H_2_O_2_ and fed with BPL1™, BPL1™ HT or LTA from BPL1™. Image taken with a Nikon-SMZ18 Fluorescence Stereomicroscope (Scale bar: 250 µm).

As shown in Figure 2A, BPL1™ cells significantly increased the *C. elegans* lifespan compared with the NGM control, with an increase in the mean lifespan of 1 day (8.33%), and in the final lifespan of 4 days (19.05%) (*p*-value < 0.0001). Likewise, heat-treated BPL1™ cells also provoked a significant positive effect on nematodes’ lifespans (*p*-value < 0.01), increasing the mean lifespan in 1 day (6.67%) and 3 days (13.04%) regarding the final lifespan (Figure 2B), while the purified LTA from BPL1™ increased the mean lifespan by 1 day (7.7%) and the final lifespan by 2 days (8.7%) (*p*-value < 0.01) (Figure 2C). These results are in line with the previously observed correlation on fat reducing activity of BPL1™ and its heat-treated form and LTA from BPL1™ [10].

To identify the underlaying mechanism of action of the pro-longevity effect of BPL1™, BPL1™ HT and LTA from BPL1™, additional lifespan experiments were performed with different *C. elegans* mutant strains of the IGF-1 (DAF-2/DAF-16) and MAPK pathways (PMK-1/p38 and JNK-1).

The positive effect on lifespan observed for BPL1™, BPL1™ HT and LTA from BPL1™ was completely lost in *C. elegans daf-16* and *daf-2* mutant strains, whereas the increase in *C. elegans* lifespan remained statistically significant in the other mutant strains analyzed, *pmk-1* and *jnk-1* (Table 1). This evidence indicates that, similar to the fat reduction activity, the positive effect on *C. elegans* lifespan was regulated by the insulin-like signaling pathway IGF-1.

Previous studies have shown an age-related increase in oxidative stress mediated by the accumulation of reactive oxygen species (ROS) that could provoke molecular damage [31,32]. As we had previously reported that BPL1™ alive cells had a significant antioxidant activity [9], then we aimed to determine whether BPL1™ HT and LTA from BPL1™ also had antioxidant effects. Through oxidative stress assays on the *C. elegans* model, we observed that both BPL1™ HT and LTA from BPL1™ showed a protective effect against oxidative stress, as a significant survival increase was determined in treated nematodes (*p*-value < 0.05) (Figure 2D). These results are consistent with other reported probiotics that maintained similar levels of antioxidant activity after heat treatment [5].

During aging, intestinal permeability increases, causing age-associated reductions in cognition or physical functions [33]. In this sense, *C. elegans* has emerged as a good model to study the gut barrier integrity using the Nile Red staining [24].

Experiments were performed to study whether BPL1™, BPL1™ HT and LTA from BPL1™ could protect against increased intestinal permeability. The addition of H_2_O_2_ (0.8 mM) to the nematodes damaged the intestine, causing the passage of dye to the body and increasing the fluorescence (30.06%) (Figure 2E). By contrast, the treatment with either BPL1™, BPL1™ HT or LTA from BPL1™ induced a significant fluorescence reduction (Figure 2F,G), suggesting that either BPL1™, BPL1™ HT or LTA from BPL1™ would help to maintain the integrity of the nematodes’ gut barrier.

### 3.3. BPL1™, Heat-Treated BPL1™ and LTA from BPL1™ Exert Protective Effect against Pathogen Infection

**Figure 3:**
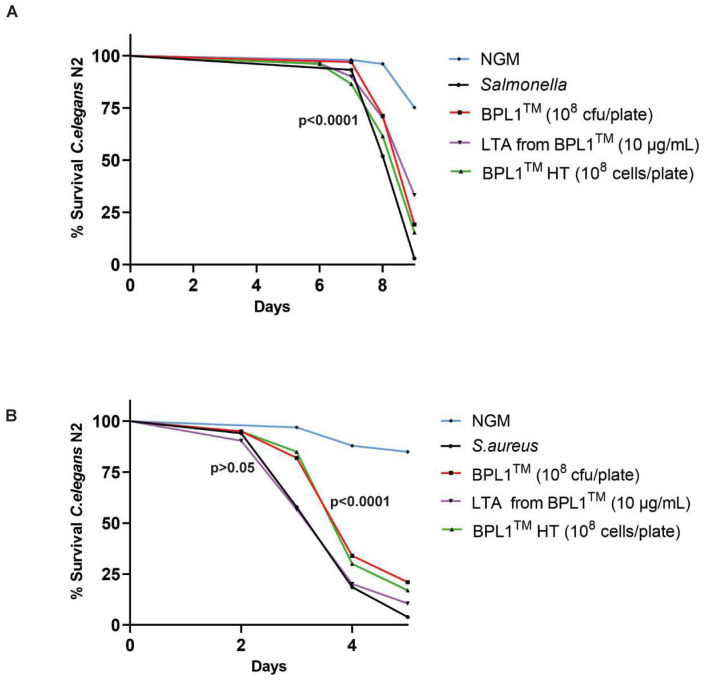
BPL1™, BPL1™ HT and LTA from BPL1™ exert protective effects against pathogen infection. (**A**) Survival rate of N2 wild-type nematodes fed with *E. coli* OP50 strain, *S. enterica* subsp. *enterica* serovar *Typhimurium* ATCC 14028 strain and BPL1™ (10^8^ cfu/plate), BPL1™ HT (10^8^ cells/plate) or LTA from BPL1™ (10 µg/mL) in the presence of the pathogen. (*p <* 0.0001 in the 3 conditions vs. infection.) (**B**) Survival rate of N2 wild-type nematodes fed with *E. coli* OP50 strain, *S. aureus* ATCC 25923 strain and BPL1™ (10^8^ cfu/plate) (*p* < 0.0001), BPL1™ HT (10^8^ cells/plate) (*p* < 0.0001) or LTA from BPL1™ (*p* > 0.05) in the presence of the pathogen. Data are the average of two independent experiments (*n* = 100/condition). Log Rank *t*-test was applied.

The BPL1™ strain and its LTA exert a positive effect on *C. elegans* lifespan and weight control through the insulin-like signaling pathway IGF-1. The IGF-1 pathway is also involved in the regulation of the defense against pathogen infections in *C. elegans* [34]. We hypothesized that BPL1™, BPL1™ HT and its LTA could have a role in protection from pathogens. In this sense, infection assays in *C. elegans* were performed with a Gram-positive (*S. aureus* ATCC 25923 strain) and Gram-negative (*S. enterica* subsp. *enterica* ATCC 14028 strain) pathogenic bacteria. As shown in Figure 3A, *S. enterica* ATCC 14028 infection provoked an increase in mortality between days 6 and 9 of infection. On the other hand, the addition of BPL1™, BPL1™ HT or LTA from BPL1™ to the nematodes’ diet counteracted this effect, and survival significantly increased during the infection period compared with the infected condition (*p*-value < 0.0001). This effect was particularly high in the case of LTA from BPL1™ at an exposure level of 10 µg/mL. In the case of *S. aureus* ATCC 25923 infection, this pathogen provoked mortality up to 80% at day 4, and nearly 100% at day 5 (Figure 3B). Conversely, nematodes fed with BPL1™ or BPL1™ HT significantly increased the survival, having 40% survival at day 4 and 20% survival at day 5 (*p*-value < 0.0001) (Figure 3B). Alternatively, infection assays performed with LTA from BPL1™ did not exert positive effect against *S. aureus* ATCC 25923 infection (Figure 3B and Figure S1A). Thus, BPL1™ and BPL1™ HT showed protection against both Gram-positive and Gram-negative pathogens, whereas LTA from BPL1™ exerted a highly protective effect against *S. enterica* ATCC 14028 infection, but no effect was observed against *S. aureus* ATCC 2592 infection.

### 3.4. BPL1™, Heat-Treated BPL1™ and Its LTA Improve C. elegans Anxiety and Stress-Related Behaviors and Had Positive Effect on C. elegans Alzheimer’s Model

Previous studies performed with the strain BPL1™ have indicated an upregulation of tryptophan synthesis after supplementation in the nematode’s diet [9], and modest improvements in some mental health parameters in children with Prader–Willi syndrome [14]. Considering this previous evidence supporting the role of BPL1™, and having demonstrated the anti-aging effect of BPL1™, BPL1™ HT and its LTA, we further assessed their role in different *C. elegans* behavior models.

**Figure 4:**
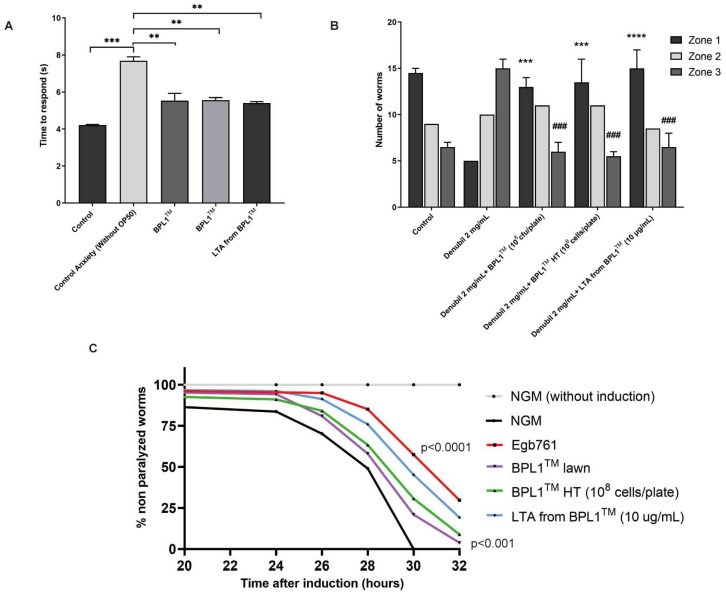
BPL1™, BPL1™ HT and LTA from BPL1™ have positive effects on anxiety-related behaviors in *C. elegans*. (**A**) Quantification of the time to respond to the octanol after applying starvation during 20 min in N2 wild-type worms fed with BPL1™ (10^8^ cfu/plate), BPL1™ HT (10^8^ cells/plate) or LTA from BPL1™ (10 µg/mL). Data are the average from two independent assays (*n* = 80/condition). *** *p* < 0.001, ** *p* < 0.01. Student’s *t*-test was applied. (**B**) Quantification of the number of N2 wild-type worms distributed in each zone after the addition of Denubil, and fed with BPL1™ (10^8^ cfu/plate), BPL1™ HT (10^8^ cells/plate) and LTA from BPL1™ (10 µg/mL) (*** *p* < 0.001, **** *p* < 0.0001). (### *p* < 0.001). Data are the average of two independent assays (*n* = 60/condition). Two-way ANOVA was applied. (**C**) Percentage of CL4176 non-paralyzed worms fed with BPL1™ overnight lawn, BPL1™ HT (10^8^ cells/plate) and LTA from BPL1™ (10 µg/mL) after induction by temperature raising. *Ginkgo biloba* extract (EGb 761) at 100 µg/mL was included as a positive control. Nematodes without temperature induction were also included as a negative control. BPL1™ (*p* < 0.001), BPL1™ HT and LTA from BPL1™ (*p* < 0.0001). Data correspond to two independent assays (*n* = 100/condition). Log Rank *t*-test was applied.

The avoidance behavior was used as an anxiety-related conduct in *C. elegans*, which is mediated by the serotonin pathway [26]. The assay quantifies the time it takes for the nematodes to start the backwards movement when they smell octanol. As previously stated [26], control anxious worms without food took longer to avoid octanol than control condition nematodes with *E. coli* OP50. However, the nematodes fed with BPL1™, BPL1™ HT or LTA from BPL1™ significantly reduced the avoidance time under food deprivation (*p*-value < 0.05), therefore counteracting the anxiety-related behavior caused by the lack of food (Figure 4A).

We also analyzed the nematode’s exploratory behavior after the addition of the psychostimulant drug Denubil. BPL1™, BPL1™ HT and LTA from BPL1™ were evaluated in this cholinergic-induced model for stress, developed in the laboratory, to test whether they are able to counteract this behavior. Figure 4B shows the distribution of worms in solid NGM plates at each feeding condition, including a non-stressed control and stressed conditions induced with Denubil. Under the non-stressed condition, nematodes tended to accumulate in the center of the plates (Zone 1), while when stressed with the Denubil, nematodes acquired an exploratory behavior with a trend to move to the edge of the plates (Zone 3). Moreover, when stress-induced nematodes were fed with BPL1™, BPL1™ HT or LTA from BPL1™, a reduction in the number of nematodes in Zone 3 was observed (*p*-value < 0.001), with a behavior pattern similar to the control (non-stressed) nematodes. Thus, BPL1™, BPL1™ HT and LTA from BPL1™ reversed the induced stress and decreased the exploratory capacity of the nematodes.

Finally, the potential benefits of BPL1™, BPL1™ HT or LTA from BPL1™ were also tested in the *C. elegans* Alzheimer’s Disease model, a well-suited model for correlating Aβ expression and toxicity by scoring the progressive paralysis phenotype in the transgenic strain CL41762 [27]. Results indicated that BPL1™, BPL1™ HT and LTA from BPL1™ provoked a reduction in the nematodes’ body paralysis when compared with control conditions (Figure 4C and Figure S1B). Particularly, LTA from BPL1™ caused a high delay (between 26 h to 32 h) in body paralysis after the induction (*p*-value < 0.0001), presenting a major positive effect compared to the live and heat-treated cells (Figure 4C). This evidences how the potential of BPL1™, BPL1™ HT and LTA from BPL1™ improves the toxicity derived from accumulation of the Aβ peptide in the nematode, which is known to trigger downstream neurotoxic events, leading to neuronal dysfunction and death [35].

Cumulatively, these results demonstrate the potential roles of BPL1™, BPL1™ HT and LTA from BPL1™ in the modulation of anxiety-related behaviors and the effectiveness of reducing the body paralysis on the *Alzheimer´s* Disease model in *C. elegans*.

## 4. Discussion

Previous studies have shown *Bifidobacterium animalis* subsp. *lactis* BPL1™ as a probiotic strain with a high fat reduction effect. This anti-obesity function was first assessed in the *Caenorhabditis elegans* model [9], and then in pre-clinical studies using Zücker rats and Wistar rats under a cafeteria diet [11,12]. Moreover, the effect of both BPL1™ and its heat-treated form was substantiated in a study with obese human volunteers [13]. Further investigations with BPL1™ conducted by our group allowed the characterization of the molecule responsible for the functional activity of the strain, the Lipotheichoic Acid (LTA) [10]. Few studies compile beneficial properties associated to the LTA of probiotic strains [36,37], but to our knowledge, LTA from BPL1™ was the first LTA described to demonstrate fat reduction properties.

Mechanism-of-action analyses with the model organism *C. elegans* showed that the fat reduction activity of BPL1™ required the FOXO transcription factor DAF-16 [9]. DAF-16 is a well-known regulator of the insulin-like signaling pathway IGF-1 in *C. elegans* [38]. Interestingly, additional research with the novel LTA from BPL1™ and the heat-treated form of the strain reveals that both act through IGF-1 to perform their fat reduction activity [10]. Moreover, in the present study, we corroborated that in the presence of BPL1™, BPL1™ HT or LTA from BPL1™, DAF-16 is activated and translocated to the nucleus. This is in accordance with the activation of DAF-16 that has been described for other *Bifidobacterium* species exerting a pro-longevity and stress tolerance effect [25].

**Figure 5:**
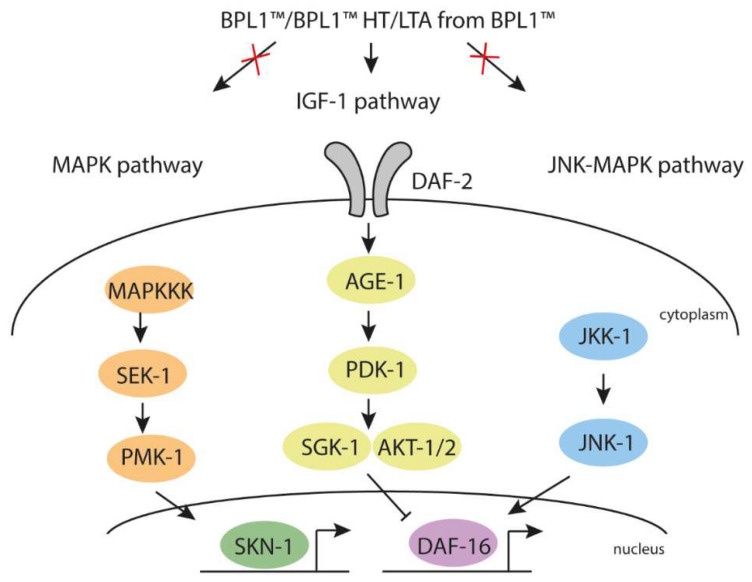
BPL1™, BPL1™ HT and the LTA from BPL1™ regulate fat deposition and longevity through the IGF-1 pathway. Schematic representation of the core components of the IGF-1, JNK-1/DAF-16 and p38 MAPK pathways.

Moreover, in this study, we explored certain alternative pathways that could act in parallel to the IGF-1 pathway. The JNK signaling pathway has been shown to act jointly with the IGF-1 pathway [39,40] and PMK-1/p38 could transmit environmental stress to DAF-16 [28]. Our results demonstrate that the IGF-1 pathway is the main metabolic target for the anti-obesity effect of BPL1™ and LTA from BPL1™ while the genes *jnk-1* and *pmk-1*, belonging to the JNK-1/DAF-16 pathway and p38 MAPK pathway, respectively, are not required. These results suggest that the fat reduction effect of BPL1™ and its LTA requires predominantly the IGF-1 pathway (Figure 5).

The IGF-1 pathway has been reported to regulate different functions such as longevity or pathogen defense, in addition to the lipid metabolism [41,42]. In fact, our results demonstrate that BPL1™, BPL1™ HT and the LTA from BPL1™ have positive effects on *C. elegans* lifespan. Few reports have shown that probiotic strains could exert longevity effects through the JNK-1/DAF-16 pathway and p38 MAPK pathway [43,44]. Likewise, as seen previously with BPL1™ fat reduction activity, the positive effect on *C. elegans* lifespan only required the IGF-1 signaling pathway.

Previous reports have shown lifespan extension due to osmotic stress, as an osmotic adaptive response [45,46]. However, addition of LTA to NGM plates did not alter osmolarity of the medium, therefore discarding a potential effect of osmotic pressure on lifespan.

The close relationship between oxidative stress and longevity has been extensively described [32]. Preceding studies with BPL1™ showed a protective effect against oxidative stress [9], and transcriptional changes in nematodes feeding on BPL1™ revealed significant upregulation of antioxidative metabolic pathways (i.e., glutathione metabolism, peroxisome) and genes (i.e., *gst-10*, *gst-39*, *gsto-1*), all involved in the response to oxidative stress. Some of these observations were validated with a metabolomic analysis in worms feeding on BPL1™, suggesting again the antioxidative effect of the strain. Furthermore, genes like *sod*-4 and *trxr*-2, involved in the maintenance of REDOX homeostasis, also play an important role in BPL1™ functionality. In this study, we validate that BPL1™ HT and LTA from BPL1™ can exert a protective effect against oxidative stress in a similar way as previously described for the BPL1™ alive cells. This activity was dependent on the insulin IGF-1-like signaling pathway and DAF-16 transcriptional factor, key regulators of oxidative stress resistance [47]. Thus, these findings are consistent with existing reports that underline the relation of oxidative stress and lipid metabolism in *C. elegans* [48].

In our work, we have also tested the potential anti-pathogenic protective role of BPL1™, BPL1™ HT and LTA from BPL1™ by analyzing the protective effect against selected Gram-positive and Gram-negative pathogens. Both BPL1™, BPL1™ HT and the LTA from BPL1™ improved viability of the nematodes during the infection with *S. enterica* ATCC 14028 strain. Likewise, BPL1™ and BPL1™ HT exerted a protective effect against *S. aureus* ATCC 25923 infection. These results are in agreement with previous reports showing a protective effect of both alive and heat-treated probiotics against pathogens [49]. On the other hand, LTA from BPL1™ did not induce a positive effect against *S. aureus* ATCC 25923 infection. Lipoteichoic acid from *S. aureus* has been described as a pivotal component in biofilm formation and infection [50]. We hypothesize that LTA from BPL1™ could be neutralized by the LTA present in the *S. aureus* ATCC 25923 strain. Further research with the BPL1™ strain will be necessary in order to elucidate if there are other molecules responsible for the protection against *S. aureus* ATCC 25923 infection.

There is increasing evidence about how gut microbiome composition could modulate the brain and behavior by means of the so-called gut–brain axis [51]. Studies with *Lactobacillus* sp. and *Bifidobacterium* sp. in aged mice and clinical trials reveal the potential of probiotics in the areas of cognitive ability, anxiety or depression [52,53]. Previous transcriptomic studies have shown that metabolic synthesis pathways of amino acids were upregulated in *C. elegans* feeding on BPL1™ [9]. Among them, tryptophan, a precursor amino acid involved in the serotonin synthesis pathway, was increased in BPL1™-fed nematodes. In turn, serotonin has been reported to have a key role in diverse functions of the *C. elegans* nervous system and other tissues like pharyngeal pumping, egg laying, male mating, regulation of locomotion and feeding behavior [54,55]. In addition, *C. elegans* has emerged as a simple model to analyze many behaviors that reveal cognitive impairment, or model aspects of various psychiatric disorders [56]. Moreover, neurotransmitters and neuropeptides are conserved by comparing the mammalian nervous system (dopamine, GABA, acetylcholine, serotonin, etc.), and the nematode has shown a remarkable behavioral plasticity, similar to learning and memory in mammals [57]. In our study, we determined a positive activity of BPL1™, BPL1™ HT and LTA from BPL1 in anxiety-associated behavior in *C. elegans.* Nematodes treated with our probiotic strain, or its LTA, show behavioral differences between anxious nematodes under deprivation of food. Similarly, nematodes submitted to a psychostimulant stress through exposure to the drug Denubil tended to move to the edge of the plates under control conditions, but nematodes fed with BPL1™, BPL1™ HT or LTA from BPL1™ presented a different behavioral pattern with nematodes staying in the center of the plate. Our results are in line with the evidence observed in a study in children with Prader–Willi syndrome, in which the administration of BPL1™ modestly improved some mental symptoms of this disease [14]. These results reveal the potential of the BPL1™ strain modulating behavioral aspects, but further studies will be required in order to validate potential of BPL1™ and its LTA in cognition.

Finally, BPL1™, BPL1™ HT and LTA from BPL1™ were evaluated in the Alzheimer’s Disease *C. elegans* model. Alzheimer’s Disease is a neurodegenerative disease that usually affects elderly people [58]. Alzheimer’s Disease pathogenesis is associated with the accumulation of the beta amyloid peptide outside the neurons [59], and evidence on the involvement of oxidation in triggering Alzheimer’s Disease has previously been reported [60]. In the Alzheimer’s Disease *C. elegans* model, the CL4176 strain expresses a muscle-specific Aβ1–42 that under a temperature shift induces the paralysis of the worms. With this strain, we demonstrate that BPL1™, BPL1™ HT and LTA from BPL1™ prevent the nematodes´ body paralysis. These results highlight the anti-aging potential of BPL1™, BPL1™ HT and the LTA from BPL1™ by exerting a positive effect on a specific neurodegenerative disease that affects mainly elderly individuals. Overall, this study investigates the targets whereby the BPL1™ strain and its LTA exert their beneficial effect. Results highlight the pivotal role of the insulin-like signaling pathway in fat reduction activity as well as the newly confirmed pro-longevity effect. Moreover, BPL1™ and its LTA show beneficial effects against oxidative stress and pathogen infection and a potential improvement in cognitive function in the *C. elegans* pre-clinical model.

## 5. Conclusions

In summary, in this study, we investigated additional metabolic pathways underlaying the functionality of the probiotic *Bifidobacterium animalis* subsp. *lactis* BPL1™, the postbiotic BPL1™HT and its LTA. Results indicate that the IGF-1 insulin pathway is the main target, thus eliminating alternative metabolic pathways such as p38 MAPK and JNK-1/DAF 16 pathways. Furthermore, results show for the first time the pro-longevity effect of BPL1™, BPL1™ HT and its LTA in *C. elegans*, also through the IGF-1 pathway. Our findings reveal an improvement in oxidative stress and gut permeability markers, together being a protection against pathogenic infections in nematodes feeding on the strain and its derivates. Finally, different results obtained in *C. elegans* behavioral models have shown the positive effect of BPL1™, the postbiotic BPL1™ HT and its LTA in counteracting anxiety-related and stress behaviors, together with an improvement in a mobility marker in the Alzheimer’s Disease model. All these results validate the functional effect of the BPL1 ™ strain (and its heat-treated version and LTA) in fat reduction, confirming the IGF-1 as the primary metabolic target. Furthermore, for the first time, we show the potential application of BPL1™ and the postbiotic version in the aging field, highlighting its functional activity in improving cognitive functions and proteotoxicity in the nematode, which should be investigated in future studies.

## 6. Patents

The patent EP3113.25 results from the work reported in this manuscript.

## Figures and Tables

**Table 1:** Effect of BPL1 and HT-BPL1 on different *C. elegans* mutant strains’ lifespan. Curve comparisons vs. *E. coli* OP50 are indicated (*p*-values). Two independent experiments were performed (*n* = 100–200/condition). Log Rank *t*-test was applied. * *p* < 0.05, ** *p* < 0.01, *** *p* < 0.001, **** *p* < 0.0001, NS: not significant.

Strain	Treatment	Worms (*n*)	Mid Lifespan (Days)	Final Lifespan (Days)	Log Rank χ^2^	ρ-Value	Significance
N2 (wild-type)	*E. coli* OP50	200	12	22			
	BPL1	200	13	25	36.6	<0.0001	****
*daf-16(mgDf50)*	*E. coli* OP50	200	13	22			
	BPL1	200	13	23	0.04417	0.83	NS
*daf-2(e1370)*	*E. coli* OP50	200	26	53			
	BPL1	200	26	54	0.666	0.4145	NS
*pmk-1(km25)*	*E. coli* OP50	200	13	22			
	BPL1	200	15	27	55.26	<0.0001	****
*jnk-1(gk7)*	*E. coli* OP50	200	10	20			
	BPL1	200	13	22	82.25	<0.0001	****
N2 (wild-type)	*E. coli* OP50	100	15	23			
	BPL1 HT	100	16	26	9.152	0.0025	**
*daf-16(mgDf50)*	*E. coli* OP50	100	12	23			
	BPL1 HT	100	12	23	1.183	0.2768	NS
*daf-2(e1370)*	*E. coli* OP50	100	18	53			
	BPL1 HT	100	19	55	0.1213	0.7276	NS
*pmk-1(km25)*	*E. coli* OP50	100	15	23			
	BPL1 HT	100	18	24	9.439	0.0021	**
*jnk-1(gk7)*	*E. coli* OP50	100	13	22			
	BPL1 HT	100	17	23	10.94	0.0009	***
N2 (wild-type)	*E. coli* OP50	200	12	23			
	LTA from BPL1	200	12	24	6.14	0.0132	*
*daf-16*(mgDf50)	*E. coli* OP50	200	13	23			
	LTA from BPL1	200	13	23	0.02458	0.76	NS
*daf-2(e1370)*	*E. coli* OP50	200	39	58			
	LTA from BPL1	200	36	60	1.533	0.2157	NS
*pmk-1(km25)*	*E. coli* OP50	200	13	26			
	LTA from BPL1	200	14	27	8.368	0.0038	**
*jnk-1(gk7)*	*E. coli* OP50	200	12	26			
	LTA from BPL1	200	15	27	9.251	0.0024	**

## Data Availability

All data generated or analyzed during this study are included in this article and its supplementary information files.

## Supplementary Materials

The following supporting information can be downloaded at: https://www.mdpi.com/article/10.3390/antiox12122107/s1, Figure S1 includes the results of the analysis of different LTA from BPL1™ doses in the pathogen infection assay of *C. elegans* (*S. aureus*) (A) and the positive dose response effect of LTA from BPL1™ in the *C. elegans* Alzheimer´s Disease model (B).
