# Supplementary material for: Bifidobacterium animalis subsp. lactis BPL1™ and Its Lipoteichoic Acid Modulate Longevity and Improve Age/Stress-Related Behaviors in Caenorhabditis elegans
Source: Antioxidants (Basel). 2023 Dec 13;12(12):2107. doi: 10.3390/antiox12122107 (PMC10740966; doi:10.3390/antiox12122107)
Supplement: Supplementary file 1 [file antioxidants-12-02107-s001.zip › antioxidants-2739978-supplementary.pdf]

Supplementary Materials

Figure S1 includes the results of the analysis of different LTA from BPL1^{TM} doses in the pathogen infection assay of *C. elegans* (*S. aureus*) (A) and the positive dose response effect of LTA from BPL1^{TM} in the *C. elegans´* Alzheimer´s Disease model (B).

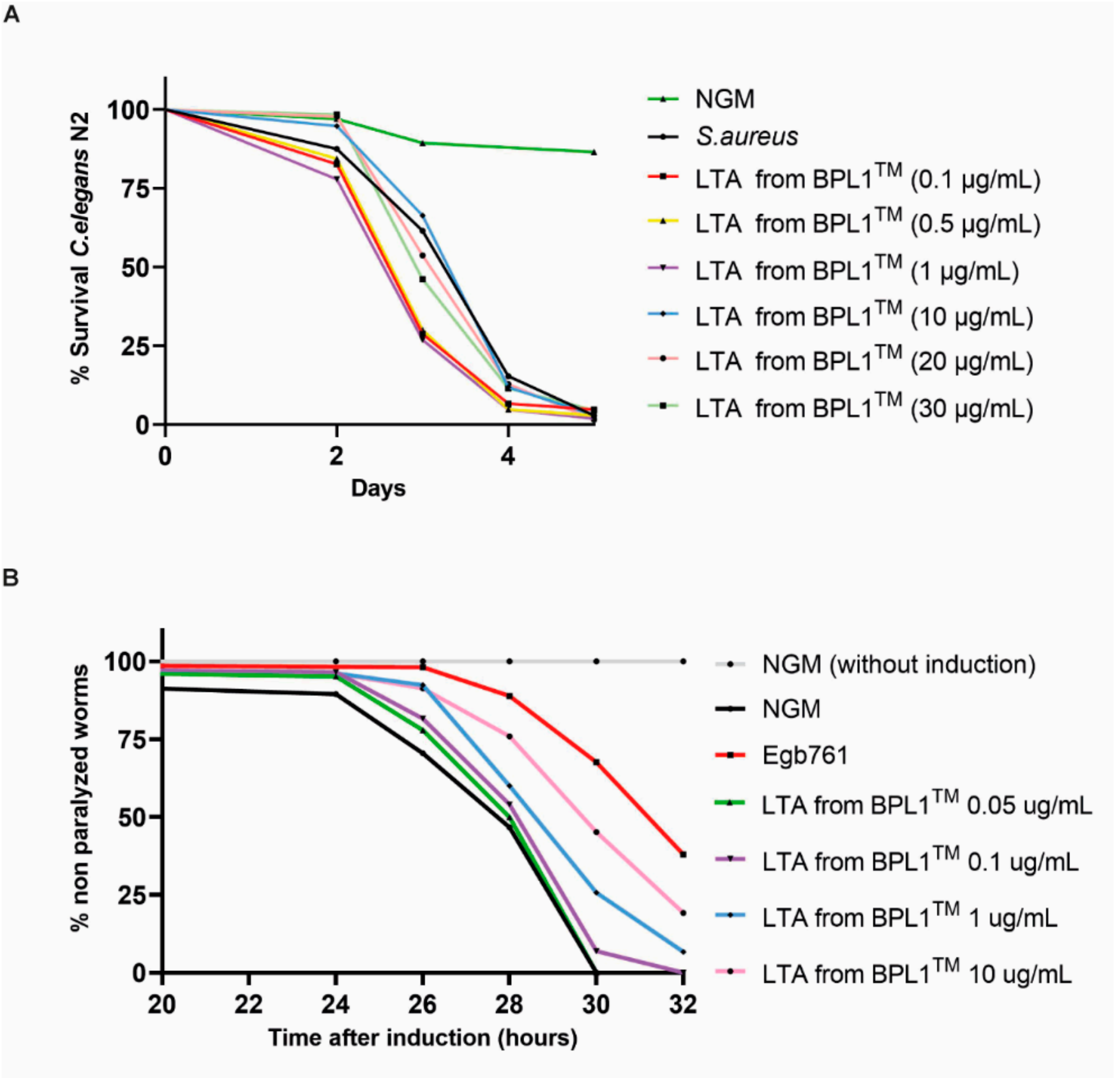

Figure S1. (A) Dose-Response of survival rate of N2 wild type nematodes fed with *E. coli* OP50 strain, *S. aureus* ATCC 25923 strain and different doses of LTA from BPL1^{TM}. No statistical differences observed. Data are the average of two independent experiments (n = 100/condition). Log Rank T-test was applied. (**B)** Percentage of CL4176 non-paralyzed worms fed different doses of LTA from BPL1^{TM}. Optimal dose 10 µg/mL ($p < 0.0001$). Data correspond to two independent assays (n = 100/condition). Log Rank *t*-test was applied.
